# Supplementary material for: Mesenchymal Stromal Cell-Derived Small Extracellular Vesicles Modulate Apoptosis, TNF Alpha and Interferon Gamma Response Gene mRNA Expression in T Lymphocytes
Source: Int J Mol Sci. 2023 Sep 5;24(18):13689. doi: 10.3390/ijms241813689 (PMC10530670; doi:10.3390/ijms241813689)
Supplement: Supplementary file 1 [file ijms-24-13689-s001.zip › Figure S1.pdf]

Figure S1. MSC Characterization

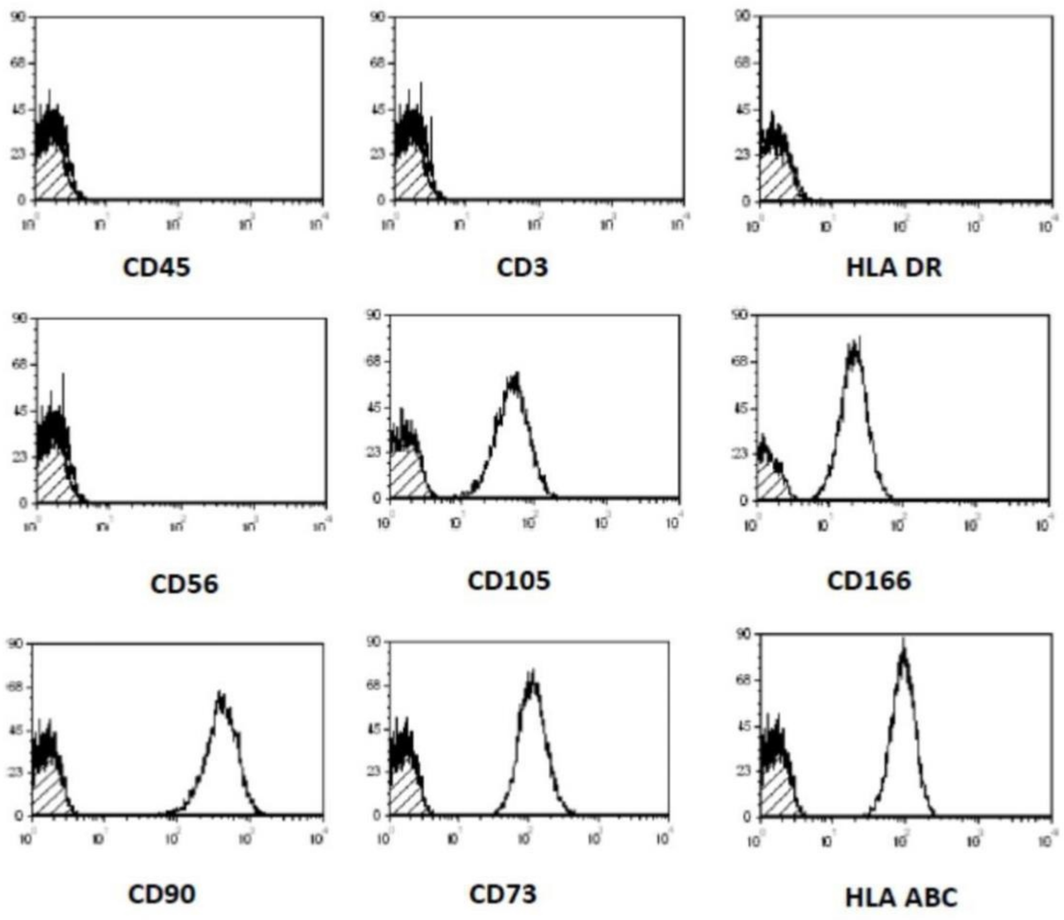

Cells' identity was confirmed by flow cytometry analysis (FACS) using established criteria with positive markers (anti CD73, CD166, CD105, HLA-ABC and CD90 antibodies) and negative markers (anti HLA-DR, CD56, CD3 and CD45 antibodies).
