# Supplementary material for: Mesenchymal Stromal Cell-Derived Small Extracellular Vesicles Modulate Apoptosis, TNF Alpha and Interferon Gamma Response Gene mRNA Expression in T Lymphocytes
Source: Int J Mol Sci. 2023 Sep 5;24(18):13689. doi: 10.3390/ijms241813689 (PMC10530670; doi:10.3390/ijms241813689)
Supplement: Supplementary file 1 [file ijms-24-13689-s001.zip › Figure S2.pdf]

**Figure S2: Electron Microscopy**

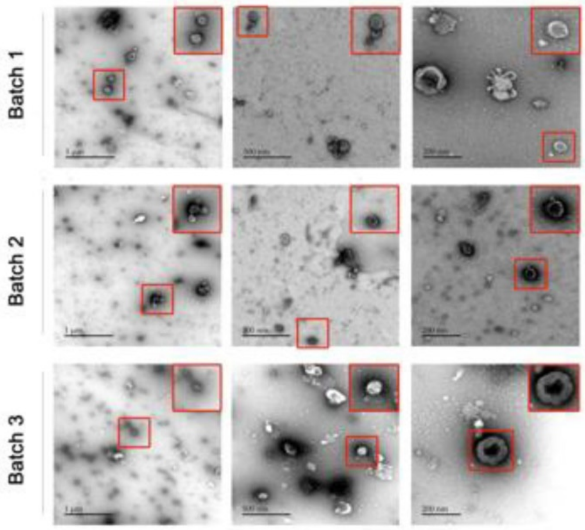


Samples were stained with Nanovan (previously vortexed for 3-5 min). Briefly, 5 µl of samples were placed on formvar/carbon coated copper 200 mesh grids (EMS), mixed with 5 µl NV (NANOVAN, Nanoprobes, NY, USA) for 5-10 sec, excess stain was blotted off and grids were dried. Grids were visualized with Jeol® JEM-1400 Plus transmission electron microscope (TEM, Jeol®, Tokyo, Japan), equipped with ORIUS SC600 CCD camera (Gatan®, Abingdon, UK), and Gatan Microscopy Suite program (DigitalMicrograph, Gatan®, UK).
