# Supplementary material for: Mesenchymal Stromal Cell-Derived Small Extracellular Vesicles Modulate Apoptosis, TNF Alpha and Interferon Gamma Response Gene mRNA Expression in T Lymphocytes
Source: Int J Mol Sci. 2023 Sep 5;24(18):13689. doi: 10.3390/ijms241813689 (PMC10530670; doi:10.3390/ijms241813689)
Supplement: Supplementary file 1 [file ijms-24-13689-s001.zip › Table S6.pdf]

Table S6: Donor characteristics. (a)**.** MSC donors' characteristics. (b). Lymphocyte donors' characteristics.

a.

**MSC Donor no.**	**Age**	**Gender**
**1**	53	Male
**2**	33	Female
**3**	23	Male

b.

**Lymphocyte Donor no.**	**Age**	**Gender**
**1**	55	Female
**2**	64	Male
**3**	32	Female
**4**	54	Male
**5**	38	Male
**6**	41	Female
**7**	31	Male
